# Whole-genome sequencing of clinical isolates of *Citrobacter Europaeus* in China carrying *bla*_OXA−48_ and *bla*_NDM−1_

**DOI:** 10.1186/s12941-024-00699-y

**Published:** 2024-04-29

**Authors:** Jie Ma, Ranran Xu, Wanxiang Li, Mi Liu, Xiaomei Ding

**Affiliations:** 1 Department of Clinical Laboratory, Weifang People’s Hospital, Weifang, Shandong China; 2 Department of Clinical Laboratory, The First Affiliated Hospital of Weifang Medical University, Weifang Medical University, Weifang, Shandong China

**Keywords:** *Citrobacter Europaeus*, Whole-genome sequencing, Carbapenem-resistance genes, *bla*_NDM−1_, *bla*_OXA−48_

## Abstract

**Objective:**

To analyze the clinical infection characteristics and genetic environments of resistance genes in carbapenem-resistant *Citrobacter europaeus* using whole-genome sequencing.

**Methods:**

The susceptibility of two clinical isolates of *C. europaeus* (WF0003 and WF1643) to 24 antimicrobial agents was assessed using the BD Phoenix™ M50 System and Kirby-Bauer (K-B) disk-diffusion method. Whole-genome sequencing was performed on the Illumina and Nanopore platforms, and ABRicate software was used to predict resistance and virulence genes of carbapenem-resistant *C. europaeus*. The characteristics of plasmids carrying carbapenem-resistance genes and their genetic environments were analyzed. Single nucleotide polymorphisms were used to construct a phylogenetic tree to analyze the homology of these two *C. europaeus* strains with ten strains of *C. europaeus* in the NCBI database.

**Results:**

The two strains of carbapenem-resistant *C. europaeus* are resistant to various antimicrobial agents, particularly carbapenems and β-lactams. WF0003 carries *bla*_NDM− 1_, which is located on an IncX3 plasmid that has high homology to the pNDM-HN380 plasmid. *bla*_NDM− 1_ is located on a truncated Tn*125*. It differs from Tn*125* by the insertion of IS*5* in the upstream ISA*ba125* and the deletion of the downstream ISA*ba125*, which is replaced by IS*26*. WF1643 carries *bla*_OXA− 48_ in a Tn*1999* transposon on the IncL/M plasmid, carrying only that single drug resistance gene. Homology analysis of these two strains of *C. europaeus* with ten *C. europaeus* strains in the NCBI database revealed that the 12 strains can be classified into three clades, with both WF0003 and WF1643 in the B clade.

**Conclusion:**

To the best of our knowledge, this is the first study to report an IncX3 plasmid carrying *bla*_NDM− 1_ in *C. europaeus* in China. *C. europaeus* strains harboring carbapenem-resistance genes are concerning in relation to the spread of antimicrobial resistance, and the presence of carbapenem-resistance genes in *C. europaeus* should be continuously monitored.

## Introduction

*Citrobacter* spp. are gram-negative bacteria belonging to the Enterobacteriaceae family. They mostly act as opportunistic pathogens and can cause diarrhea, sepsis, meningitis, or respiratory and urinary tract infections in infants, young children, and immunocompromised patients [1, 2]. Currently, *Citrobacter* spp. are rapidly gaining clinical importance as multidrug-resistant pathogens causing opportunistic hospital- acquired and community-acquired infections.

In recent years, the excessive usage of antimicrobial agents, including carbapenems, has led to an increase in bacterial antimicrobial resistance [3]. In the first half of 2023, Enterobacteriaceae were still the primary pathogenic microorganisms causing hospital-acquired infections in China, according to data from CHINET (http://www.chinets.com/). Specifically, *Citrobacter* spp. showed resistance rates to meropenem, imipenem, ceftazidime, and cefoxitin of 6.1%, 5.4%, 25.9%, and 51.9%, respectively. At present, the emergence of carbapenem-resistant *Citrobacter* has increased the rate of hospital-acquired infections and mortality [4, 5]. Acquisition of carbapenem resistance by opportunistic pathogens poses a serious challenge to the treatment of infections. Moreover, it highlights the importance of monitoring antimicrobial-resistant bacteria along with investigation of resistance mechanisms.

Mechanisms of carbapenem resistance mainly include carbapenemase production and high production of AmpC enzymes or extended-spectrum β-lactamase, along with the deletion of outer-membrane pore proteins and/or high expression of efflux pumps [6]. The importance of carbapenemases is greater because most of their coding genes are carried on mobile genetic elements such as plasmids and transposons that can be transferred between bacteria [6]. The New Delhi metallo-β-lactamase *bla*_NDM−1_ is the most common carbapenem-resistance gene in Enterobacteriaceae, which is mainly located on plasmids. Plasmids carrying *bla*_NDM−1_ are diverse in the Enterobacteriaceae family, and the most common include IncX, IncA/C, IncF, and IncL/M incompatibility plasmids [7–10]. Mobile genetic elements—such as transposons, insertion sequences, integrons, and other movable genetic elements surrounding the structure of *bla*_NDM−1_ in Enterobacteriaceae—mediate the transmission of *bla*_NDM−1_ among bacteria [11]. *bla*_OXA−48_ is a globally prevalent carbapenem-resistance gene [12]. Tn*1999* and efficient transfer of multiple plasmids (such as pOXA-48a, accession number JN626286) have accelerated the horizontal spread of *bla*_OXA−48_ among bacteria [13]. Therefore, the study of the structure of plasmids carrying carbapenem-resistance genes and their genetic environments can help in understanding the mechanism of widespread distribution of carbapenem-resistance genes in pathogens.

The aim of this study was to analyze the clinical infection characteristics and genetic environments of resistance genes in carbapenem-resistant *Citrobacter europaeus* in our hospital in China.

## Methods

### Bacterial isolation and identification

Two carbapenem-resistant *C. europaeus* strains (WF0003 and WF1643) were isolated from hospitalized patients at a tertiary hospital in northern China. WF0003 was isolated from ascites in 2011, and WF1643 was isolated from sputum in 2017. The strains were identified by Matrix Assisted Laser Destruction/Ionization Time of Flight Mass Spectrometry (MALDI-TOF MS) using Vitek MS (Sysmex bioMerieux, Marcy l’Etoole, France).

### Antimicrobial susceptibility testing

The BD Phoenix™ M50 System (Becton, Dickinson and Company, New Jersey, USA) and K-B (K-B) disk-diffusion method (Oxoid, Hampshire, United Kingdom) was used to test the susceptibility of the strains to 24 antimicrobial agents commonly used in clinical practice. The susceptibility criteria were determined in accordance with the Clinical and Laboratory Standards Institute (CLSI) guidelines (2021) [14] (https://clsi.org/). *Escherichia coli* ATCC 25,922 served as the quality control strain.

### Whole-genome sequencing and sequence assembly

The bacterial genomes were extracted using a bacterial DNA kit (OMEGA, GA, USA), and the purified DNA was subjected to whole-genome sequencing using a combination of the Illumina HiSeq X Ten (Illumina, CA, USA) and Nanopore PromethION (Oxford Nanopore Technologies, OX, UK) sequencing platforms. Paired-end DNA libraries were constructed with an average insert size of 350 bp for Illumina sequencing, and shotgun DNA libraries were generated with a 10 kb insert size for Nanopore sequencing. FastQC 0.11.8 (http://www.bioinformatics.babraham.ac.uk/projects/fastqc/) was used for quality control of raw reads. A hybrid assembly was then performed using Unicycler v0.4.9 [15], incorporating both the short paired-end Illumina reads and the long Nanopore reads.

### Whole-genome sequencing analysis

Antimicrobial-resistance genes and virulence genes were identified using the NCBI and Virulence Factors of Pathogenic Bacteria (VFDB) databases with ABRicate 0.8 (https://github.com/tseemann/abricate) [16]. PlasmidFinder [17] was used to examine the type of plasmid replicon. The sequences of other strains were obtained using NCBI for the constructing of the phylogenetic tree (up to Sept 30, 2023). Then, the core single nucleotide polymorphisms (SNPs) were identified by Mummer 3.25 [18]. Maximum-likelihood phylogenetic trees were constructed using MEGAX 10.1.8 [19] based on the resulting core SNPs with a bootstrap iteration of 1000, and visualized by Interactive Tree Of Life (iTOL) [20]. Annotation of mobile elements, and other features utilized online databases, including ISfinder [21], and Tn Number Registry [22]. DANMEL was used for analyzing the sequenced mobile genetic elements in bacteria [23]. BLASTN was employed for the alignment and comparison of the plasmid sequences analyzed in this study with high homologous plasmid sequences publicly available in NCBI. Gene organization diagrams were drawn in Inkscape 0.48.1 software.

### Nucleotide sequence accession numbers

The complete sequences of the two *C. europaeus* strains were submitted to GenBank under BioProject number PRJNA1088829.

## Results

### Patient background and clinical isolates of *C. europaeus*

Patient 1 was a 62-year-old female with chronic hepatitis B who was hospitalized in the infectious unit, and WF0003 was isolated from her ascites. The patient was treated with cefepime (for four days) and imipenem (for two days). The patient was discharged from the hospital in a poor state.

**Fig. 1:**
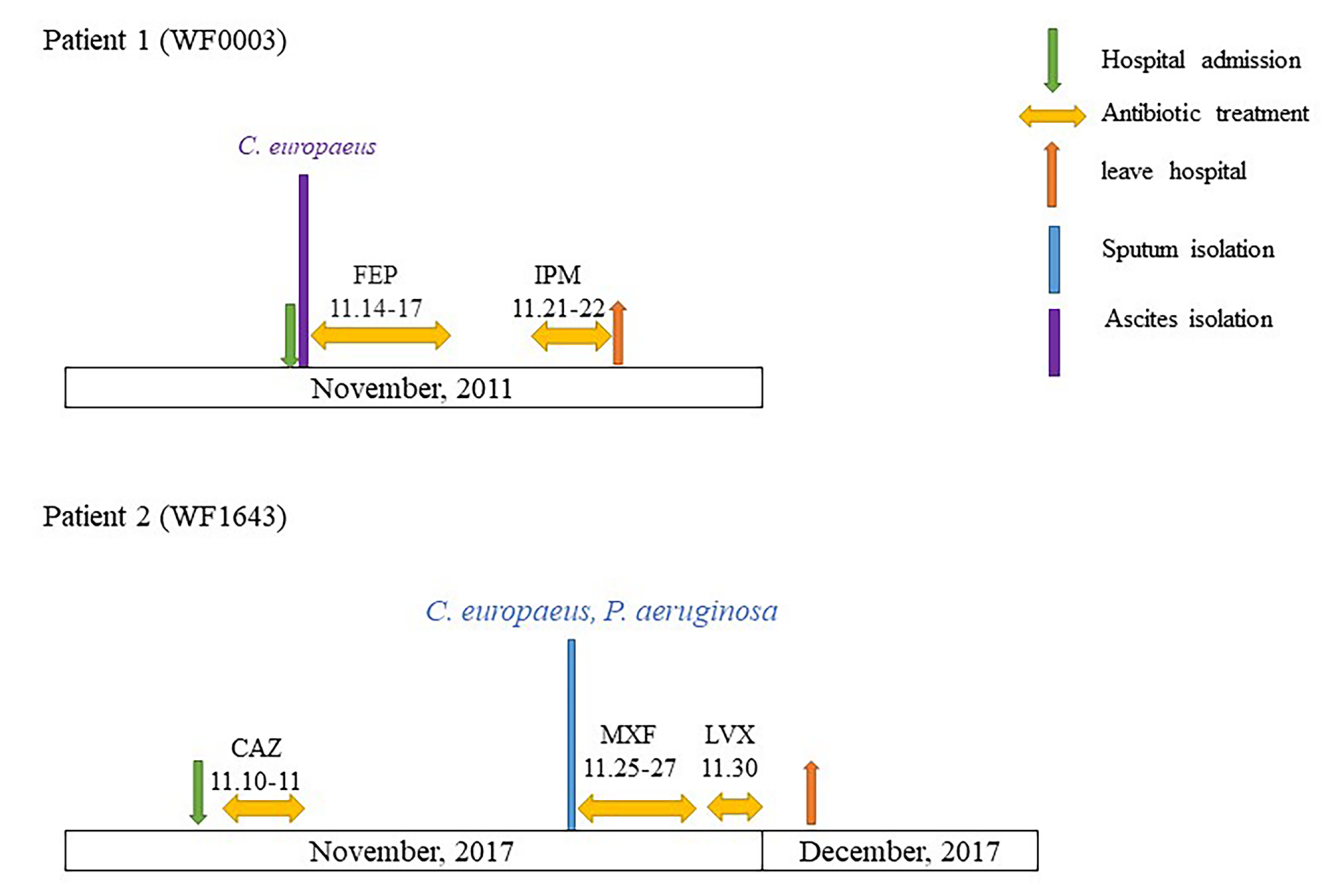
Antimicrobial agents used during hospitalization

Patient 2 was an 87-year-old male with basal cell carcinoma of the face who was admitted from the healthcare setting, and WF1643 was isolated from his sputum. The patient received ceftazidime (for two days) before the WF1643 isolation, and was treated with moxifloxacin (for three days) and levofloxacin (for a day) after the WF1643 isolation. The patient was discharged from the hospital with an improvement in health. The hospital treatments of patients 1 and 2 are shown in Fig. 1.

### Antimicrobial susceptibility testing

The results of the antimicrobial susceptibility testing revealed that the two strains of *C. europaeus* have high resistance rates to antimicrobial agents (Table 1). WF0003 and WF1643 are resistant to 13 and 10 antimicrobial agents, respectively. Both strains of *C. europaeus* exhibit high resistance to β-lactams and are susceptible to only three antimicrobial.

**Table 1:** Case characteristics and antimicrobial susceptibility test results of the two *C. europaeus* strains

Antimicrobial agents		Method	WF0003	WF1643
β-lactam/β-lactamase inhibitor combinations	AMC	MIC(µg/ml)	> 32/R	> 32/R
	TZP	MIC(µg/ml)	> 64/R	> 64/R
	SAM	MIC(µg/ml)	16/I	16/I
	ATM	MIC(µg/ml)	> 32/R	16/R
	CAZ	MIC(µg/ml)	> 32/R	32/R
	CRO	MIC(µg/ml)	> 32/R	> 32/R
	CXM	MIC(µg/ml)	16/I	16/I
	CZO	MIC(µg/ml)	> 16/R	> 32/R
	FEP	MIC(µg/ml)	> 16/R	≤ 1/S
	FOX	MIC(µg/ml)	16/I	16/I
	CTX	K-B(mm)	6/R	18/R
	AMP	K-B(mm)	6/R	6/R
Quinolones	LVX	K-B(mm)	24/S	18/I
	CIP	K-B(mm)	22/I	28/S
Carbapenems	ETP	MIC(µg/ml)	> 2/R	> 2/R
	IMP	MIC(µg/ml)	> 8/R	4/R
	MEM	MIC(µg/ml)	> 8/R	1/S
Sulfonamides	SXT	MIC(µg/ml)	> 4/R	≤ 1/S
Polymyxins	COL	MIC(µg/ml)	≤ 1/S	≤ 1/S
Tetracyclines	TGC	MIC(µg/ml)	≤ 1/S	≤ 1/S
chloromycetin	C	MIC(µg/ml)	16/I	8/S
Aminoglycosides	AMK	MIC(µg/ml)	≤ 8/S	≤ 8/S
	GEN	MIC(µg/ml)	8/I	≤ 2/S
	TOB	MIC(µg/ml)	8/I	≤ 2/S

CRO, ceftriaxone; AMK, amikacin; GEN, gentamicin; AMP, ampicillin; ATM, aztreonam; CAZ, ceftazidime; CIP, ciprofloxacin; LVX, levofloxacin; FEP, cefepime; IPM, imipenem; ETP, ertapenem; SXT, trimethoprim-sulfamethoxazole; TOB, tobramycin; TZP, piperacillin-tazobactam; SAM, ampicillin-sulbactam; MEM, meropenem; CXM, cefuroxime; CZO, cefazolin; CTX, cefotaxime; CSL, cefoperazone/sulbactam; C, chloramphenicol; AMC, amoxicillin-clavulanic acid; COL, colistin; TGC, tigecycline

### Resistance and virulence genes

WF0003 has 23 resistance genes, which are mainly against β-lactams and aminoglycosides, including the carbapenem-resistance gene *bla*_NDM−1_ and the polymyxin-resistance gene *mcr-9*. WF1643 has three resistance genes, including the carbapenem-resistance gene *bla*_OXA−48_ (Table 2).

Twenty-five and 27 virulence genes were identified in WF0003 and WF1643 isolates, respectively, which the most common were Vi-antigen-related, adhesion-related, nutrient/metabolism-related, and yersiniabactin-encoding genes (Table 2).

**Table 2:** Predicted resistance and virulence genes of *C. europaeus*

Isolates NO.	Resistance genes	Virulence genes
WF0003	*mcr-9, tet(D), catA2, bla*_DHA−1_, *qnrB4, sul1, ere(A), aac(3)-II, aac(6’)- II c, mph(A), bla*_SFO−1_, *aadA2, dfrA12, aac(3)- IId, bla*_TEM−1_, *bla*_SHV−12_, *ble*_MBL_, *bla*_NDM−1_, *dfrA19, aph(3’’)-Ib, aph(6)-Id, qnrB33, bla*_CFE−1_	*tviB tviC tviD tviE vexA vexB vexC vexD vexE fliG csgB csgD csgE csgF csgG ybtX ybtQ ybtP ybtA fyuA ompA entB entE fepC chuX*
WF1643	*qnrB33, bla*_CFE−1_, *bla*_OXA−48_	*tviB tviC tviD tviE vexA vexB vexC vexD vexE fliG csgB csgD csgE csgF csgG ybtX ybtQ ybtP ybtA fyuA ompA entA entB entE entS fepC chuX*

### Genome features of the plasmids carried by *C. europaeus*

Three types of plasmids (IncHI2, IncX3, and RepA_1) were found in WF0003 (Table 3). pWF0003-1 is an IncHI2-type plasmid carrying multiple drug-resistance genes, including *mcr-9*. pWF0003-NDM plasmid is a 54-kb IncX3-type plasmid, carrying three resistance genes: *bla*_NDM−1_, *bla*_SHV−12_, and *ble*_MBL_. Furthermore, a 62-kb IncL/M-type plasmid (pWF1643-OXA) carrying *bla*_OXA−48_ was identified in WF1643.

**Table 3:** Genomic features of *C. europaeus*

Category	WF0003			WF1643
	pWF0003-1	pWF0003-NDM	pWF0003-3	pWF1643-OXA
Incompatibility group	IncHI2	IncX3	RepA_1	IncL/M
Total length(bp)	338,497	54,035	13,411	62,812
Total number of ORFs	361	43	19	102
Mean G + C content,%	48.3	49.0	50.7	51.2
Resistance gene	*mcr-9, tet(D), catA2, bla*_DHA−1_, *qnrB4, sul1, ere(A), aac(3)-II, aac(6’)- II c, mph(A), bla*_SFO−1_, *aadA2, dfrA12, aac(3)- II d, bla*_TEM−1_	*bla*_SHV−12_, *ble*_MBL_, *bla*_NDM−1_	*dfrA19, aph(3’’)-Ib, aph(6)-Id*	*bla*_OXA−48_

### Analysis of genetic environments of carbapenem-resistance genes

**Fig. 2:**
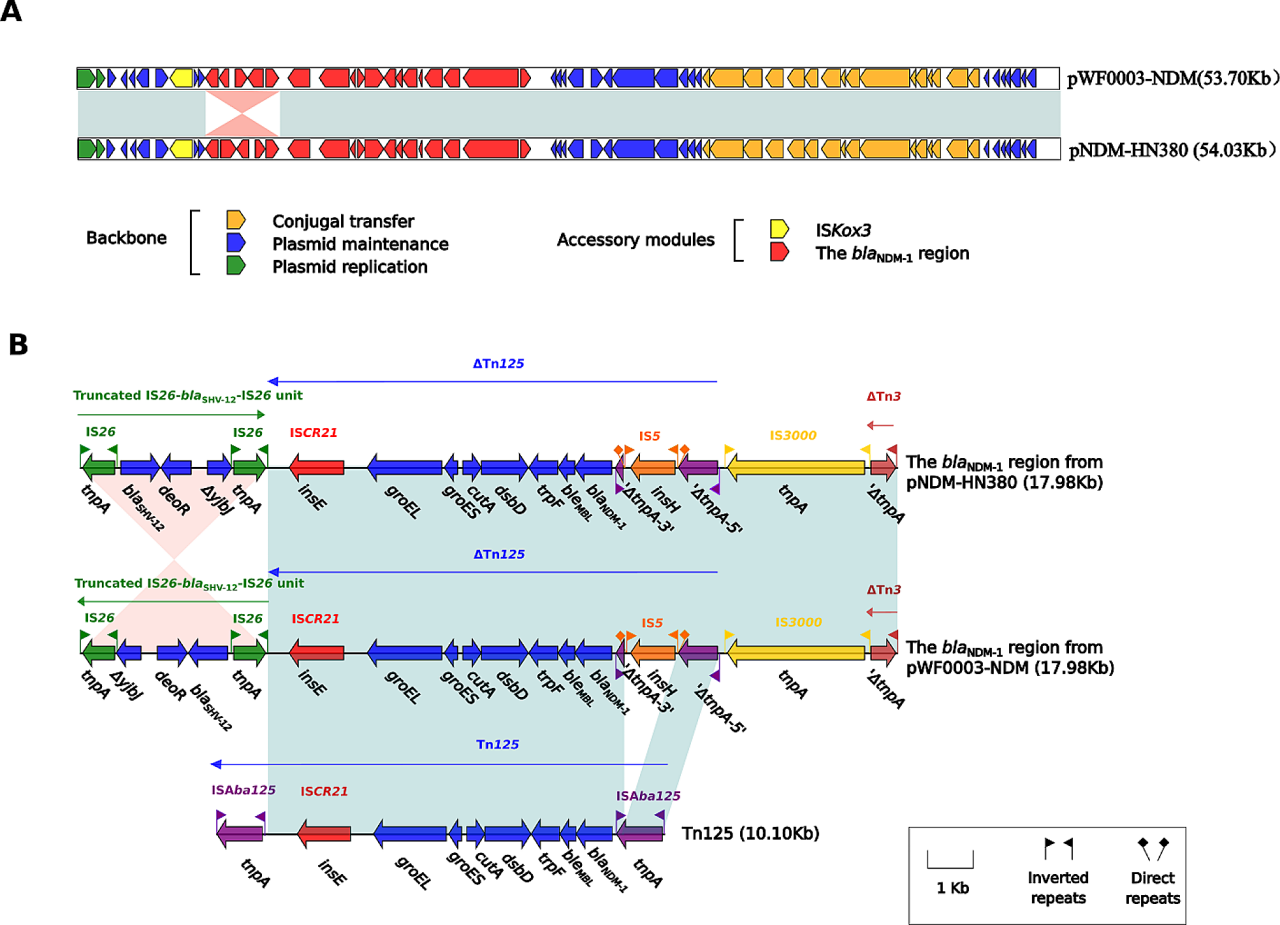
Comparison of plasmid pWF0003-NDM with pNDM-HN380. (**A**) Linear comparison of two sequenced pWF0003-NDM plasmids with pNDM-HN380. (**B**) The *bla*_NDM−1_ region from pWF0003-NDM and comparison with related regions

The pWF0003-NDM plasmid carrying *bla*_SHV−12_, *ble*_MBL_, and *bla*_NDM−1_ genes is 99% homologous to pNDM-HN380 (GenBank accession no. JX104760) (Fig. 2A). An inversion of the truncated IS26-blaSHV-12-IS26 unit in the *bla*_NDM−1_ region represents the only modular difference between pWF0003-NDM and pNDM-HN380. *bla*_NDM−1_ is located on ΔTn*125*. ΔTn*125* on pWF0003-NDM differs from Tn*125* in that the upstream ISA*ba125* has an IS*5* insertion, and the downstream ISA*ba125* is missing and replaced by IS*26*. *bla*_SHV−12_ is located in the truncated IS*26*-*bla*_SHV−12_-IS*26* sequence (Fig. 2B).

**Fig. 3:**
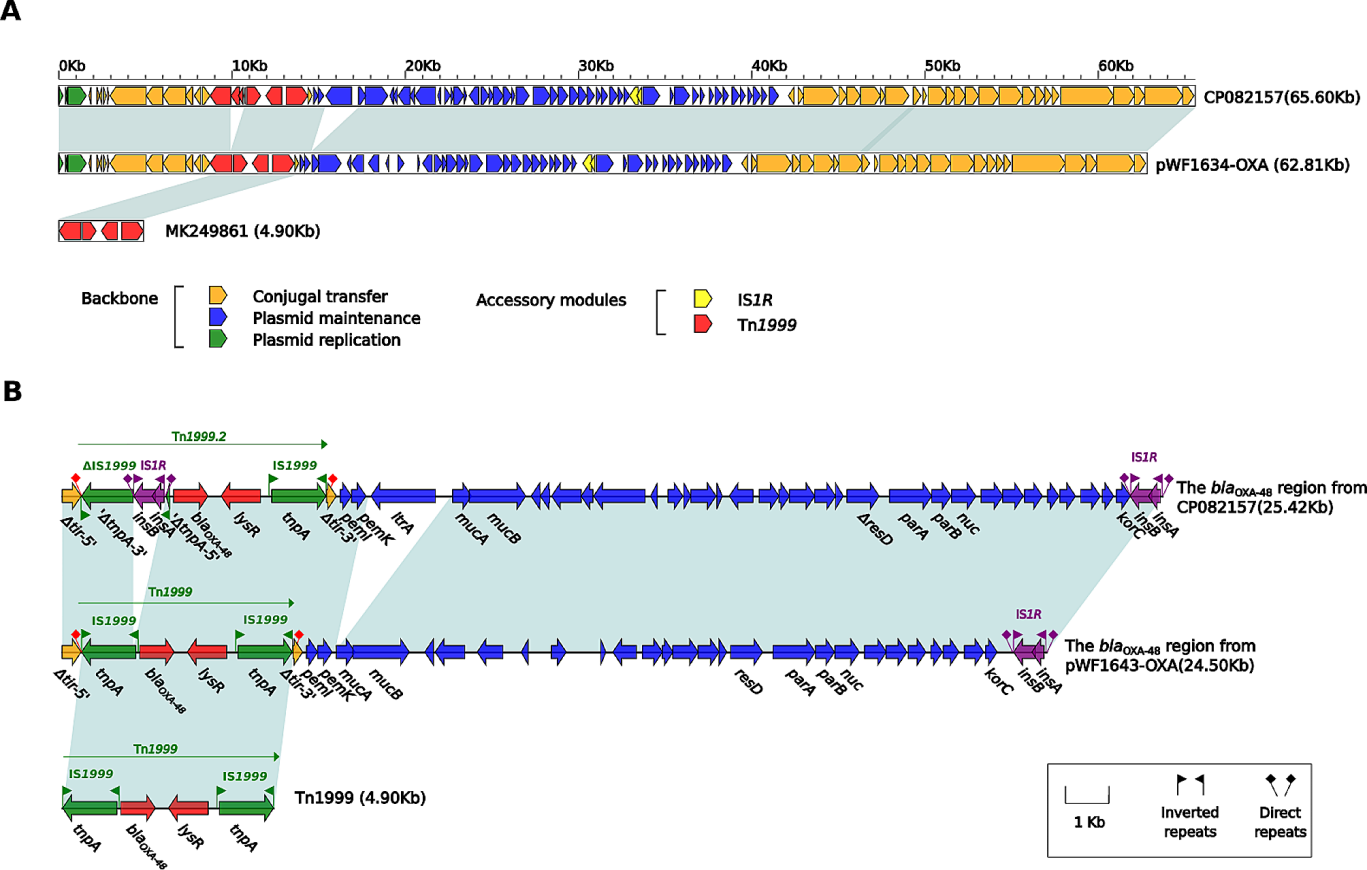
Comparison of plasmid pWF1643-OXA with CP082157. (**A**) Linear comparison of two sequenced pWF1643-OXA plasmids with CP082157. (**B**) The *bla*_OXA−48_ region from pWF1643-OXA and comparison with related regions

The pWF1643-OXA plasmid backbone region is highly homologous to pCP082157 (Fig. 3A). *bla*_OXA−48_ is located on an intact Tn*1999* flanked by IS*1999* (Fig. 3B). This transposon is inserted in the *tir* gene. The IS*1R* sequence is inserted in IS*1999* upstream of Tn*1999.2* in pCP082157. Compared with pCP082157, pWF1643-OXA (a part of the backbone region) is devoid of the Group-II intron reverse transcriptase/maturase functional gene *ltrA*.

### Homology analysis

**Fig. 4:**
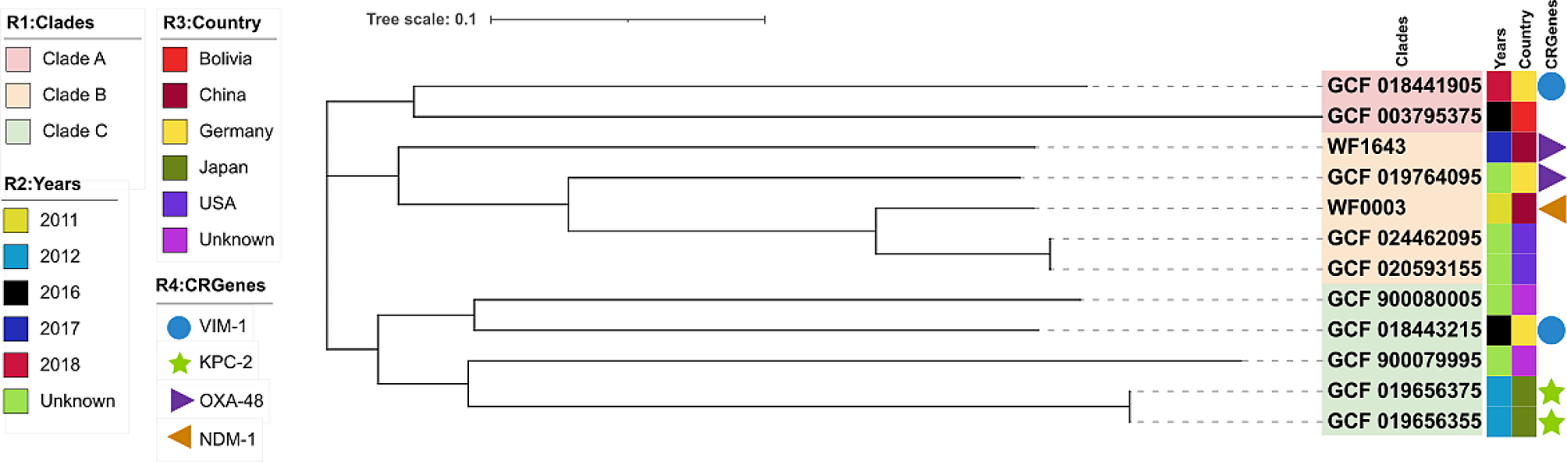
Homology analysis of *C. europaeus*

A phylogenetic tree was constructed using the two *C. europaeus* strains isolated in this study and ten strains of *C. europaeus* obtained from NCBI. This divided the 12 strains into three clades, with both strains isolated in this study in the B clade (Fig. 4). The 12 strains of *C. europaeus* were isolated from five different countries between 2011 and 2018. Two of them are from unknown sources, and the time of isolation was not reported. Four carbapenem-resistance genes (*bla*_VIM−1_, *bla*_KPC−2_, *bla*_OXA−48_, and *bla*_NDM−1_) were detected in seven of the 12 *C. europaeus* strains. WF0003 and WF1643 are closely related to *C. europaeus* strains from the USA and Germany, respectively.

## Discussion

*Citrobacter* spp. are widespread in natural environments, including soil, water, air, and food. Normally, they do not cause disease in animals and humans, and therefore their clinical pathogenicity is often overlooked [24]. Despite the low prevalence of *Citrobacter* infections, invasive *Citrobacter* are associated with a high mortality rate [25]. In this study, two *C. europaeus* strains were isolated from elderly patients with underlying diseases. After being admitted to hospital, the patients were treated with antimicrobial agents, but patient 1 was in a poor condition and ceased treatment. The results are similar to Oyeka’s study [26], in which *Citrobacter* infections were associated with serious consequences in immunocompromised patients. *C. europaeus* was first reported in 2017 by Ribeiro et al. It was named *C. europaeus* with average nucleotide identity (ANI) values significantly below the 95% species threshold of *C. freundii*, *C. werkmanii*, and *C. youngae* [27]. *Citrobacter* is easily confused with *E. coli* and *Salmonella* because it exhibits great variability in its morphological and biochemical characteristics, antigenicity, and pathogenicity [28, 29]. Previously, the identification of *Citrobacter* spp. was mainly carried out through genotypic and phenotypic approaches. Nowadays, MALDI-TOF MS technology is widely used for bacterial identification, as it is faster and more accurate than traditional methods [30, 31]. Few complete genomes of *C. europaeus* are available at NCBI, and information about this bacterium is generally little; therefore, the incidence of infection with carbapenem-resistant *C. europaeus* is likely to be underestimated. To the best of our knowledge, this is the first study to investigate carbapenem-resistant *C. europaeus* clinical isolates in our hospital. We studied the resistance mechanisms and the plasmids carrying the resistance genes to understand the possible resistance and transmission mechanisms.

In Biez’s study, they isolated 1121 carbapenem non-susceptible *Citrobacter* spp. with high-level resistance to quinolones, β-lactams, and aminoglycosides [32]. Carbapenem-resistant *C. europaeus* exhibited resistance to multiple antimicrobial agents. This is consistent with our study, in which WF0003 and WF1643 exhibited resistance to 13 and 10 antimicrobial agents, respectively. Both strains were only sensitive to three antimicrobial agents, AMK, COL, and TGC. Such a high level of resistance is of concern and may lead to the ineffectiveness of available antimicrobial agents against this pathogen.

*bla*_NDM-1_ is the most prevalent carbapenem-resistance gene in Enterobacteriaceae in China [33], and the spread of *bla*_NDM-1_ between bacteria is mainly mediated by plasmids. In this study, we have shown that WF0003 carries *bla*_NDM-1_ on an IncX3 plasmid that is similar to pNDM-HN380 (GenBank accession no. JX104760), isolated from *Klebsiella pneumonia* in Hong Kong in 2011 [34]. The pNDM-HN380 plasmid has been reported in *C. freundii*, *E. coli*, and *Enterobacter hormaechei* [35–37], suggesting that plasmids harboring resistance genes are likely to be transmitted horizontally between different strains. Although IncX3 plasmids carrying *bla*_NDM-1_ have been frequently reported in other *Citrobacter* species [35, 38], this is the first report in *C. europaeus* in China.

In this study, *bla*_NDM−1_ in WF0003 isolate is located on ΔTn*125*, the major transposon responsible for the widespread dissemination of the *bla*_NDM−1_ gene. In *Acinetobacter*, Tn*125* was identified in the pNDM-BJ01 plasmid as a composite transposon based on two identically oriented ISA*ba125* insertion sequences [39]. As per the sequencing data, Tn*125* consists of ISA*ba125*, *bla*_NDM−1_, *ble*_MBL_, *trpF*, *dsbD*, *cutA*, *groES*, *groEL*, IS*CR21*, and ISA*ba125*, and is constrained at both ends by a 3-bp DR [37]. In Enterobacteriaceae, as a result of complex recombination events, Tn*125* usually exists in various truncated forms [40]. These ΔTn*125* forms are similar to the Tn*125* variants found in several regions of China [41]. *bla*_NDM−1_ found in clinical or environmental isolates or isolates of animal origin, in China and abroad, exhibits various truncated forms of Tn*125*, although in different genetic environments [40, 42, 43]. This suggests that Tn*125* plays an important role in the transmission of *bla*_NDM−1_.

In this study, we have shown that *bla*_OXA−48_ in the WF1643 isolate is located on an IncL/M plasmid on Tn*1999*. *bla*_OXA−48_ is the most prevalent carbapenem-resistance gene in *Citrobacter* spp [32], and was discovered and characterized in a carbapenem-resistant *K. pneumonia* isolate from a Turkish patient [44]. Subsequently, this gene has been identified and reported worldwide, including in China, the United States, and India [45]. In consistent with our study, *bla*_OXA−48_ is most commonly found in the 60–70 kb IncL/M plasmid, which has no other resistance genes. *bla*_OXA−48_ is found between two IS*1999* insertion sequences on the Tn*1999* complex transposon, upstream of *lysR*. *bla*_OXA−48_ transfer is mainly carried out through a 62-kb IncL/M plasmid (pOXA-48) [46], which is found in most *bla*_OXA−48_-positive Enterobacteriaceae members. The CP082157 reference plasmid harbors the *bla*_OXA−48_ gene, which is located on Tn*1999.2*, a variant of the Tn*1999* transposon. A previous study [47] detected 91.8% Tn*1999.2* and only 8.2% complete Tn*1999*, indicating that Tn*1999.2* is more common than Tn*1999*. Potron et al. reported that IncL/M is an efficient carrier of resistance genes, and inactivation of the *tir* gene (encoding a transfer repressor protein) by the insertion of Tn*1999* may contribute to the efficient transfer of pOXA-48a in various genetic backgrounds. Moreover, the current spread of *bla*_OXA−48_ is largely a result of the spread of a single prevalent plasmid [48].

WF0003 carries *mcr-9* genes, but it is susceptible to colistin. This is consistent with Ju’s study, which isolated nine strains of *Citrobacter* carrying *mcr-9*, all of which were sensitive to colistin [49]. Kieffer’s study showed that the expression of *mcr-9* could be induced by subinhibitory concentrations of colistin in the presence of *qseB* and *qseC*. Therefore, the minimal inhibitory concentration (MIC) levels of colistin were increased [50]. This suggests that the clinical use of colistin may induce colistin resistance in *mcr-9*-positive isolates and accelerate the dissemination of *mcr-9* among potential pathogens. Therefore, the presence of *mcr-9* in pathogens should be carefully monitored.

WF1643 is similar to a *C. europaeus* strain from Germany in that they both carry *bla*_OXA−48_. The prevalence of *bla*_OXA−48_ is geographically specific, mainly concentrated in the Middle East and Europe [51]. WF1643 shares high similarity with European strains. Therefore, great attention should be paid to the prevalence and evolution of *bla*_OXA−48_ to prevent large-scale epidemic outbreaks. WF0003 is similar to two strains of *C. europaeus* from the USA, although the two strains from the USA do not carry carbapenem-resistance genes and WF0003 is the only strain that carries *bla*_NDM−1_. However, because of the small number of clinical isolates of *C. europaeus* and small amount of data uploaded to the NCBI database, the estimation of the possession of carbapenem-resistance genes in *C. europaeus* may not be truly accurate. Future studies should focus on the identification of isolates and biological analysis of *C. europaeus* to better understand clinical infections and transmission of *C. europaeus*.

The limited number of samples in this study may not be representative of the overall diversity of *C. europaeus* in this region; however, this study still provides important clues for the classification and characterization of *C. europaeus*. This study reveals the predicted virulence and resistance genes of the *C. europaeus* strains isolated in our hospital, and the plasmids and genetic environments in which the carbapenem-resistance genes are located. Moreover, to the best of our knowledge, this is the first study to report an IncX3 plasmid carrying *bla*_NDM−1_ in *C. europaeus* in China. These results are of great importance for the in-depth understanding of carbapenem resistance in *C. europaeus*.

## Data Availability

The sequences of the two C. europaeus strains were submitted to GenBank under BioProject PRJNA1088829.
